# MRI-Based Assessment of Intralesional Delivery of Bone Marrow-Derived Mesenchymal Stem Cells in a Model of Equine Tendonitis

**DOI:** 10.1155/2016/8610964

**Published:** 2016-09-26

**Authors:** Alexandra Scharf, Shannon P. Holmes, Merrilee Thoresen, Jennifer Mumaw, Alaina Stumpf, John Peroni

**Affiliations:** ^1^Department of Large Animal Medicine, College of Veterinary Medicine, University of Georgia, Athens, GA 30602, USA; ^2^Department of Biological and Agricultural Engineering, College of Engineering, University of Georgia, Athens, GA 30602, USA; ^3^Veterinary Biosciences and Diagnostic Imaging, College of Veterinary Medicine, University of Georgia, Athens, GA 30602, USA

## Abstract

Ultrasound-guided intralesional injection of mesenchymal stem cells (MSCs) is held as the benchmark for cell delivery in tendonitis. The primary objective of this study was to investigate the immediate cell distribution following intralesional injection of MSCs. Unilateral superficial digital flexor tendon (SDFT) lesions were created in the forelimb of six horses and injected with 10 × 10^6^ MSCs labeled with superparamagnetic iron oxide nanoparticles (SPIOs) under ultrasound guidance. Assays were performed to confirm that there were no significant changes in cell viability, proliferation, migration, or trilineage differentiation due to the presence of SPIOs. Limbs were imaged on a 1.5-tesla clinical MRI scanner postmortem before and after injection to determine the extent of tendonitis and detect SPIO MSCs. Clusters of labeled cells were visible as signal voids in 6/6 subjects. Coalescing regions of signal void were diffusely present in the peritendinous tissues. Although previous reports have determined that local injury retains cells within a small radius of the site of injection, our study shows greater than expected delocalization and relatively few cells retained within collagenous tendon compared to surrounding fascia. Further work is needed if this is a reality* in vivo* and to determine if directed intralesional delivery of MSCs is as critical as presently thought.

## 1. Introduction

The incidence of athletic, overuse injury continues to rise with the popularity of recreational and competitive sports in both human and veterinary patients. At this time, over 50 US clinical trials investigating the effects of biological therapeutics including platelet-rich plasma or stem cells on tendon or ligament injury are active or have recently been completed in humans (ClinicalTrials.gov). A comprehensive review of tendinopathies in the equine and human athlete has shown striking similarities and concluded that the horse provides a robust preclinical model for translational therapies [1]. The use of mesenchymal stem cells (MSCs) for tendon therapy in the horse has shown encouraging results, including superior tissue organization, composition, and mechanics compared to untreated controls [2–6]. Direct, intralesional injection of MSCs under ultrasound guidance is held as the benchmark for MSCs therapy in tendonitis [3, 4, 6–8], although little is known about the efficacy of this delivery technique.

Current tracking studies rely heavily on postmortem histological validation [9–11] or utilize low resolution imaging modalities such as nuclear scintigraphy [12, 13] and low field magnetic resonance imaging [14, 15]. Such studies report low cell retention and survival in tendon following injection of MSCs, reporting <25% cells totally retained after the first 24 hours [12, 13] and fewer than 5% of the original bolus after 10 days as confirmed by histology [11]. However, little is known about the discrete localization of cells after injection or their ability to migrate into injured tissues over time.

This study represents part of an effort to establish a model of tendon injury that can be paired with nanotechnology-based cell tracking methods to track MSCs following ultrasound-guided injection into damaged tissues [16]. Superparamagnetic iron oxide nanoparticles (SPIOs) have the ability to image and monitor cells using MRI. SPIOs are nontoxic at low concentrations and biodegradable and do not emit ionizing agents and are readily endocytosed by adherent cells in culture [17–20]. At this time, SPIOs have safely been implemented as an intracellular label for stem cell studies in the liver [21], heart [22], spinal cord and brain [19, 23], and articular cartilage [24] to study spatial distribution and migration after implantation using MRI over periods of time ranging from weeks to months [18, 25–27].

The major aims of this study were to validate the safety of labeling equine BM-derived MSCs with SPIOs and to investigate the immediate distribution of cells following ultrasound-guided, intralesional injection of MSCs into an established model of iatrogenic, flexor tendon injury in the horse [28, 29]. This model was chosen to reflect the environment associated with acute tendon injury and provide reproducible areas of tissue contrast on MRI that could be used to enhance intralesional detection of SPIOs. The hypotheses of this research were that (i) equine BM-MSCs would be unaltered by SPIO labeling, (ii) SPIOs labeled MSCs could be tracked immediately after injection in an equine tendonitis model, and (iii) MSCs would be localized within the tendon lesion following ultrasound-guided injection. This study represents the first effort to track cells in an iatrogenic model of tendon injury on a high field, clinical scanner with potential for translation into longitudinal studies of experimental and naturally occurring disease models.

## 2. Materials and Methods

### 2.1. *In Vitro* Validation

All experiments were performed in triplicate using cryopreserved, bone marrow-derived equine mesenchymal stem cells from 3 horses below passage 6. Cells were cryopreserved in 10% (v/v) DMSO in cell culture medium and thawed for 1 minute in a 37°C water bath. Cells were diluted in 10 mL MSC culture medium (low glucose Dulbecco's Modified Eagle Medium (DMEM), 10% Fetal Bovine Serum, 1% L-glutamine, 50 U/mL penicillin, and 50 *μ*g/mL streptomycin), centrifuged, and plated for culture at 10,000 cells/cm^2^. Cells were maintained at 37°C and 5% CO_2_ in MSC culture medium. At 70% confluency, MSCs were treated with 25 *μ*g/mL Molday ION C6Amine (Biopal, Inc.) suspended in 0.1 mL/cm^2^ MSC culture medium for 4 or 16 hours, as noted. Untreated cells were used as a control. Following treatment, cells were trypsinized, centrifuged, and manually counted for use in the following assays.

#### 2.1.1. Cell Viability following SPIO Treatment

For cell viability, cells were labeled with SPIOs as described above for 4 or 16 hours. Cells were harvested and evaluated immediately after treatment and 24 hours following completion of treatment. A Trypan Blue (Cellgro®) exclusion assay was performed for viability according to the manufacturer's protocol. Results were analyzed with one-way analysis of variance (ANOVA) and Dunnett's multiple comparisons test using untreated cells as the control. Bonferroni's multiple comparison test was used to compare cells immediately after treatment to cells after 24 hours of recovery.

#### 2.1.2. Iron Content and Cell Proliferation

For qualitative assessment, cells were fixed with 4% paraformaldehyde over ice for 10 minutes, incubated for 10 minutes with Prussian Blue reagents (Biopal, Inc.), and Prussian Blue-positive, intracellular deposits of SPIOs were confirmed with light microscopy. For quantitative assessment, cells were harvested, counted, and digested in dilute aqueous nitric acid for analysis by inductively coupled plasma-mass spectrometry with a VG Plasmaquad 3 (VG Instruments) to determine iron content. To measure proliferation, cells were plated in flat bottom, 96-well plates and the CyQuant® (Life Technologies) assay was performed according to the manufacturer's microplate protocol and analyzed at 0, 24, 48, and 72 hours. Results were analyzed with two-way ANOVA using Dunnett's multiple comparisons test and untreated cells as the control.

#### 2.1.3. Trilineage Differentiation

For osteogenic and adipogenic differentiation experiments, SPIO-labeled cells were plated in flat bottom, 96-well plates at a density of 28,000 cells/cm^2^ and cultured in MSC culture medium for 24 hours. Fresh MSCs (undifferentiated) were included as controls for all experiments. Osteogenic induction cultures were replenished with Hyclone® AdvanceSTEM™ osteogenic medium every 2-3 days for 28 days. Osteogenic differentiation was determined using Calcium Liquicolor® Test (Stanbio) according to the manufacturer's protocol. Calcium was extracted from the differentiated cultures with 0.6 N HCl overnight at 4°C. The supernatant was combined at a ratio of 1 : 20 in an equal portion mixture of the color and base reagents from the Calcium Liquicolor Test and read on a plate reader at 550 nm (Biotek Synergy 4).

Adipogenic induction cultures were replenished with Hyclone AdvanceSTEM adipogenic medium for 14 days and then switched to an adipogenic medium modified from Vidal et al. (2006) consisting of DMEM, 10% FBS, 5% rabbit serum, 0.5 *μ*M dexamethasone, 60 *μ*M indomethacin, 0.5 mM IBMX, 1 *μ*M insulin, 50 U/mL penicillin, and 50 *μ*g/mL streptomycin for the remaining 14 days with medium changes every 2-3 days [30]. Adipogenic cells were fixed with 4% paraformaldehyde for 10 minutes over ice and stained with Oil Red O to identify lipid deposits.

For chondrogenic differentiation, 100,000 cells/well were plated in conical bottom, 96-well plates, centrifuged for 10 minutes, and replenished with Hyclone AdvanceSTEM chondrogenic medium every 2-3 days for 28 days. Chondrogenic pellets were fixed with methanol, stained with 0.2% Alcian Blue in 0.1 M HCl overnight, extracted with 0.1 mL 6 M guanidine/HCl for 2 hours, and read at 650 nm in a plate reader (Biotek Synergy 4).

#### 2.1.4. Scratch Test

The scratch test was modified from the protocol outlined by Liang et al. [31]. Cells were plated at confluency in a 24-well plate. A p200 pipet tip was used to scratch a line through the cells. Light microscopy images were taken at 0, 8, 16, and 24 hours. Image analysis was performed using ImageJ. Distance was measured as number of pixels between 3 sets of cells per well per time point. Results were analyzed with two-way ANOVA repeated measures analysis and Bonferroni's multiple comparisons test at a significance level of 0.05.

#### 2.1.5. Inflammatory Modulation and Cytokine Production

Equine peripheral blood mononuclear cells (PBMCs) were obtained by collecting 60 mL of peripheral blood from a healthy horse into a syringe with ethylenediaminetetraacetic acid (EDTA) as an anticoagulant. Blood was layered onto Histopaque-1077 and centrifuged at 20°C for 30 min, and the PBMC layer was removed by aspiration. Cells were washed twice in phosphate buffered saline (PBS), resuspended in monocyte media (RPMI-1641 with 10% equine serum, 50 U/mL penicillin, and 50 *μ*g/mL streptomycin) at 4 × 10^6^/mL, and plated. PBMCs were incubated for 2 hours, after which nonadherent cells were washed off and adherent PBMCs were harvested for the following assays.

To assess the ability of MSCs to modulate the inflammatory response, 100,000 MSCs were plated in 12-well transwell plates and allowed to adhere for approximately 12 hours. At this time, MSC media were exchanged for 1.5 mL monocyte media/well. Next, 400,000 monocytes were added to each upper transwell in 0.5 mL monocyte media supplemented with 50 ng/mL* E. coli* LPS and allowed to incubate for 16 hours overnight. Monocytes stimulated with LPS were used as control. Results of monocytes without LPS are not reported. Supernatant was collected and used for analysis by ELISA for production of interleukin-10 (IL-10, Abcam®) and prostaglandin E_2_ (PGE_2_, Enzo® Life Sciences) according to manufacturer's protocols. An ELISA for tumor necrosis factor-*α* (TNF-*α*) was performed as previously described by Sun et al. [32]. ELISA plates were coated with anti-equine TNF-*α* polyclonal antibody overnight, washed, and incubated with samples. Plates were washed again, incubated with anti-equine TNF-*α* biotin-labeled polyclonal antibody, washed, incubated with avidin-horseradish peroxidase, washed again, incubated with a peroxidase substrate (ABTS®), and read at 405 nm on a plate reader (Biotek Synergy 4).

### 2.2. MRI Study

MRI was performed using a Siemens Symphony with TIM technology 1.5 T MRI unit with B17 software. All imaging was performed with limb centered in a 15-channel knee coil with receiver bandwidth of 130 kHz. Proton density- (PD-) weighted turbo spin-echo (TSE), true fast imaging with steady-state free precession (TRUFI) sequences, T2^*∗*^, and multiecho spin-echo (MSE) T2-weighted sequences were acquired for cell phantoms and all subjects (Table 1). Analysis was performed with Osirix DICOM software and ImageJ.

#### 2.2.1. *In Vitro* Phantom Study

A preliminary study was performed to establish the sensitivity and limits of MRI detection of SPIO-labeled MSCs. Cell phantoms were prepared by suspending 0.01, 0.1, 0.25, 0.5, and 1 × 10^6^ cells in 200 *μ*L of 1% agar in the wells of a 96-well plate. Mean signal intensities (MSI) of labeled cells were measured using a circular ROI of 8.44 mm^2^ on 4 contiguous slices acquired from TRUFI images. Signal-to-noise ratio (SNR) was calculated by dividing the MSI by the standard deviation of the background noise. A second study was performed in normal tendon. Tissue was collected from 1 horse euthanized for purposes unrelated to this study. Beginning proximally, 1, 5, 10, and 20 × 10^6^ cells were serially injected into the SDFT and scanned as described above.

#### 2.2.2. *In Vivo* MRI Analysis of Intralesional Cell Injection

All work performed in this study was done in accordance with the University of Georgia Institutional Animal Care and Use committee guidelines. All horses were scheduled to be euthanized for reasons unrelated to this project.

*(1) Pilot Lesion.* An iatrogenic lesion was made in the forelimb of one horse scheduled for anesthesia and subsequent euthanasia. While under general anesthesia, the metacarpal region was circumferentially clipped and aseptically prepared. The limb was desensitized with a ring block performed using 10 mL of 2% lidocaine infused subcutaneously just distally to the carpus. A 1 cm incision was made on the caudal aspect of the metacarpus and into the SDFT just above the proximal extent of the digital flexor tendon sheath. A 4.5 mm Steinmann pin was inserted within the SDFT and advanced 5 cm 5 times and withdrawn and the skin sutured. The horse was euthanized under general anesthesia. 10 × 10^6^ SPIO-labeled MSCs were injected into the lesion under ultrasound guidance and imaged as described above.

*(2) Model of Tendon Injury.* Unilateral SDFT lesions were created in the forelimb of six horses. A protocol was modified from Schramme et al. [28] with horses in a standing position. Horses were premedicated with i.v. flunixin meglumine (1.1 mg/kg) and then sedated with a combination of detomidine hydrochloride (10 *μ*g/kg) and butorphanol (20 *μ*g/kg) administered intravenously. The limb was surgically prepped as described above. A 1 cm incision was made on the caudal aspect of the metacarpus and into the SDFT just above the proximal extent of the digital flexor tendon sheath. While holding the limb off the ground, a 4.5 mm Steinmann pin was inserted within the SDFT and advanced 5 cm. The pin was extracted and replaced with a 5 mm arthroscopic burr. The burr was activated at 2500 rpm and inserted and withdrawn within the SDFT 5 times. The skin incision was closed with surgical staples and the forelimb was bandaged. Horses were maintained on stall rest and walked by hand two times per day and subsequently euthanized 10 days following induction of the lesion. Cell injections and MRI were performed immediately following euthanasia and removal of the limb.

Cryopreserved, BM-derived equine MSCs below passage 10 were thawed and plated 3–5 days prior to treatment for culture as described above. The day prior to imaging, cells were treated with 25 *μ*g/mL C6Amine Molday ION suspended in MSC culture media for 4 hours. At the time of injection, cells were harvested and 10 × 10^6^ cells were counted. Cells were suspended in 0.25 to 0.75 mL PBS for injection, depending on the size of the lesion as determined by MRI and ultrasound.

Immediately following euthanasia, the injured forelimb was placed in a 15-channel knee coil with the palmar side up and imaged on a Siemens 1.5 T MRI scanner. PD images were acquired in the sagittal and transverse planes and TRUFI was acquired in the dorsal plane prior to injection as described in Table 1. Next, the limb was removed from the scanner and placed on a flat surface in a horizontal position, with palmar side up for evaluation by ultrasound. This position was chosen to mimic cell injections performed on a non-weight-bearing limb, with minimal tension on the flexor tendons so as to permit the largest volume of fluid within the core lesion. Cells were delivered via a 20-gauge needle placed into the lesion under ultrasound guidance using a 7.5 mHz probe connected to a Micromaxx Ultrasound System (SonoSite, Inc., 21919 30th Drive SE, Bothell, WA 98021, USA). The cell bolus was not delivered unless the tip of the needle could be verified within the core of the lesion on transverse and longitudinal planes. Following injection, limbs were returned to MRI and scanned with all sequences described in Table 1.

The extent of hypointense signal or signal void associated with SPIO-labeled MSC dispersion was measured using Osirix software on isotropic TRUFI images proximally and distally to the site of injection. The depth of signal into the subcutaneous tissue surrounding normal tendon was also measured. Relative pixel intensity (RPI) was measured on every other slice in T2^*∗*^ transverse images from the most proximal and distal aspects where signal voids could be observed. Histograms were generated on ImageJ to quantify the RPI from the distal aspect of the suspensory to the palmar surface of the limb. Histograms were also generated from the SDFT and deep digital flexor tendons (DDFT), which were subsequently subtracted from the total RPI to quantify the amount of RPI that was likely associated with SPIOs in the paratendinous fascia and subcutaneous tissues. Pixel values >100 were excluded from analysis.

#### 2.2.3. Histology

Following MRI, 3.5–4.0 cm of affected area of the SDFT was excised at the site of injection, embedded in OCT compound (Tissue-Tek®), and longitudinally sectioned on a cryostat (Leica) at 12 *μ*m. Sections were mounted, fixed with 4% paraformaldehyde, incubated with Prussian Blue reagents (Biopal, Inc.) to evaluate the presence of iron nanoparticles, and counterstained with Nuclear Fast Red to visualize tissue morphology with light microscopy.

### 2.3. Statistical Analysis

Results were analyzed with one-way ANOVA and Bonferroni's multiple comparison tests at a significance level of 0.05 using Prism 6 software, unless otherwise noted above. Cells isolated from 3 horses were analyzed at each time point with measurements performed in triplicate. Error is reported in figures as standard error (SE) of the mean.

## 3. Results

### 3.1. *In Vitro* Validation

#### 3.1.1. Cell Viability following SPIO Treatment

**Figure 1:**
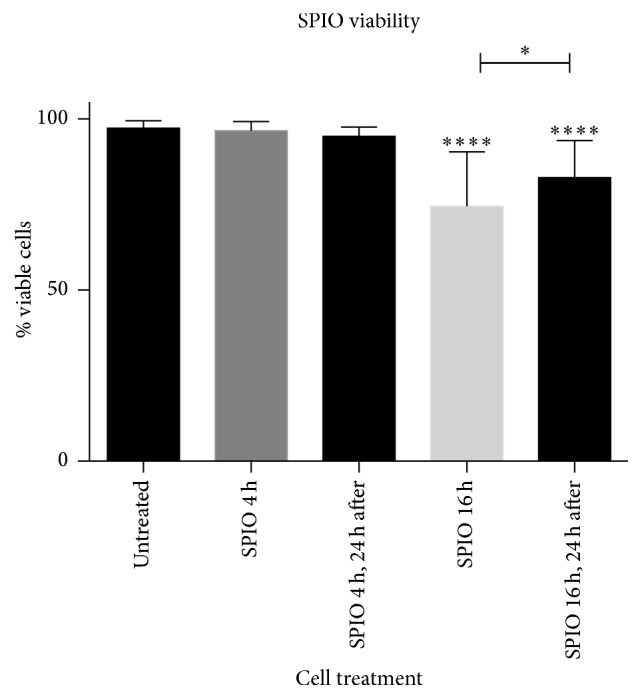
*Cell Viability*. Shown after labeling with SPIOs for 4 or 16 hours as compared to untreated cells. Measurements were recorded immediately and 24 hours after treatment (^*∗∗∗∗*^ *p* < 0.0001, ^*∗*^ *p* < 0.05).

Untreated MSCs and MSCs treated for 4 hours showed 97.35 ± 0.36 (SE) and 96.56 ± 0.64% viability, respectively (Figure 1). Viability of equine MSCs treated for 4 hours did not differ at 24 hours following treatment (Figure 1). MSCs showed a significant decrease in viability following treatment for 16 hours (74.53 ± 4.11%, *p* < 0.0001). Cells treated for 16 hours demonstrated an increase in viability 24 hours later (82.92 ± 2.55%) as compared to viability immediately following treatment (*p* < 0.05).

#### 3.1.2. Iron Content and Cell Proliferation

**Figure 2:**
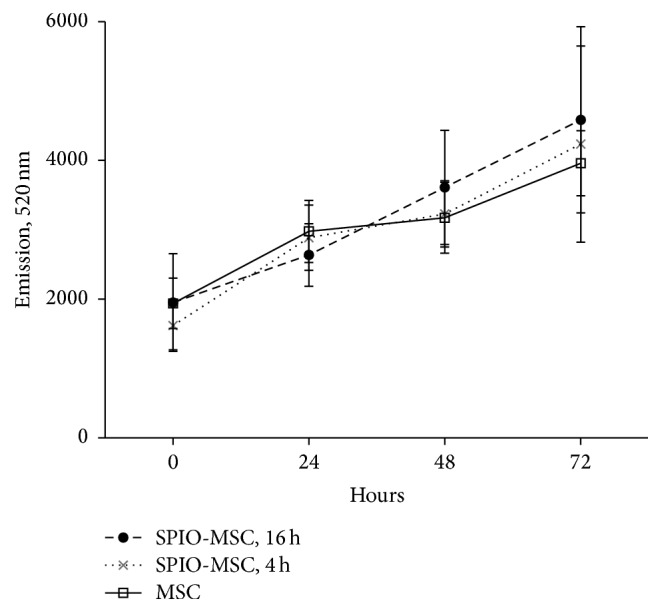
Cell proliferation shown over 72 hours after labeling with SPIOs for 4 or 16 hours as compared with untreated cells.

Iron content was determined to be 3.99 ± 0.35 (SE) and 18.64 ± 1.25 (SE) pg/cell in cells treated for 4 and 16 hours, respectively. Untreated and treated MSCs demonstrated an increasing, linear relationship proliferative capacity over 72 hours after treatment. Cell proliferation was not significantly different in untreated cells as compared to cells treated for 4 or 16 hours (Figure 2).

#### 3.1.3. Trilineage Differentiation

**Figure 3:**
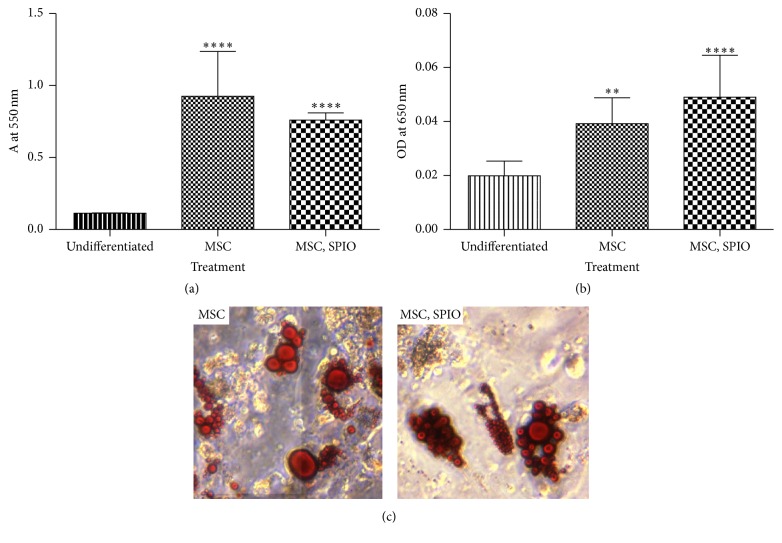
Trilineage differentiation data demonstrating (a) osteogenesis as measured by calcium production (^*∗∗∗∗*^ *p* < 0.0001), (b) chondrogenesis as measured by Alcian Blue uptake in cell pellets (^*∗∗*^ *p* < 0.01, ^*∗∗∗∗*^ *p* < 0.0001), and (c) adipogenesis as noted by lipid deposition visualized with Oil Red O.

Due to the significant difference in viability between cells labeled for 4 versus 16 hours, only cells treated for 4 hours were used in the remaining experiments. Untreated and treated cells successfully demonstrated osteogenic (*p* < 0.0001) and chondrogenic differentiation (*p* < 0.01 untreated, *p* < 0.0001 treated) as compared to undifferentiated control cells (Figures 3(a) and 3(b)). Additionally, treated and untreated cells both demonstrated adipogenic differentiation as assessed by Oil Red O staining of lipid vacuoles at 28 days after treatment (Figure 3(c)).

#### 3.1.4. Scratch Test

**Figure 4:**
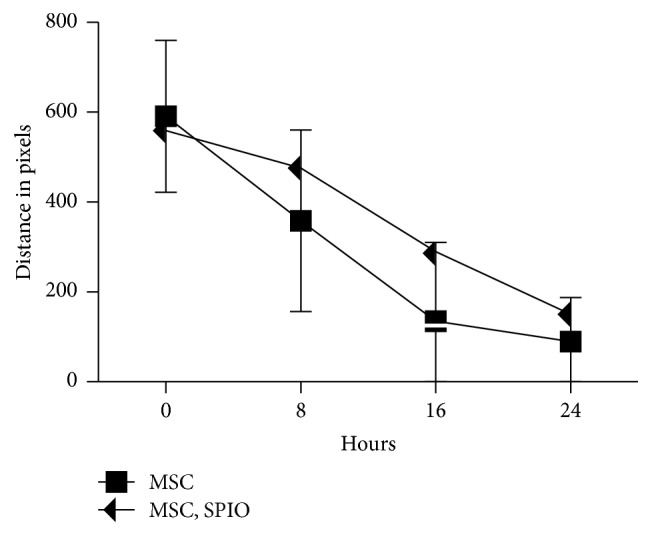
Cell migration as measured by a scratch test over 24 hours.

The scratch test showed no significant difference in the ability of treated and untreated MSCs to close the distance of the wound gap over 24 hours (Figure 4).

#### 3.1.5. Inflammatory Modulation and Cytokine Production

**Figure 5:**
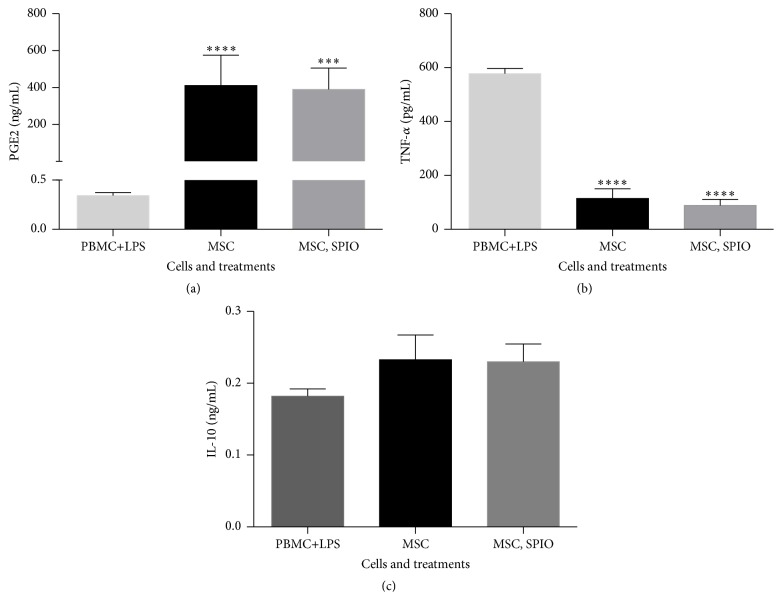
Graphs representative of (a) prostaglandin-E2 (PG-E2), (b) tumor necrosis factor-*α* (TNF-*α*), and (c) interleukin-10 (IL-10) production in MSC coculture with peripheral blood mononuclear cells (PBMCs) following stimulation with LPS (^*∗∗∗∗*^ *p* < 0.0001, ^*∗∗∗*^ *p* < 0.01).

Treated and untreated MSCs successfully upregulated PGE_2_ (*p* < 0.0001), downregulated TNF*α* (*p* < 0.0001), and upregulated IL-10 production (ns) when cocultured with PBMCs stimulated with LPS (Figure 5).

### 3.2. MRI Study

#### 3.2.1. *In Vitro* Study

**Figure 6:**
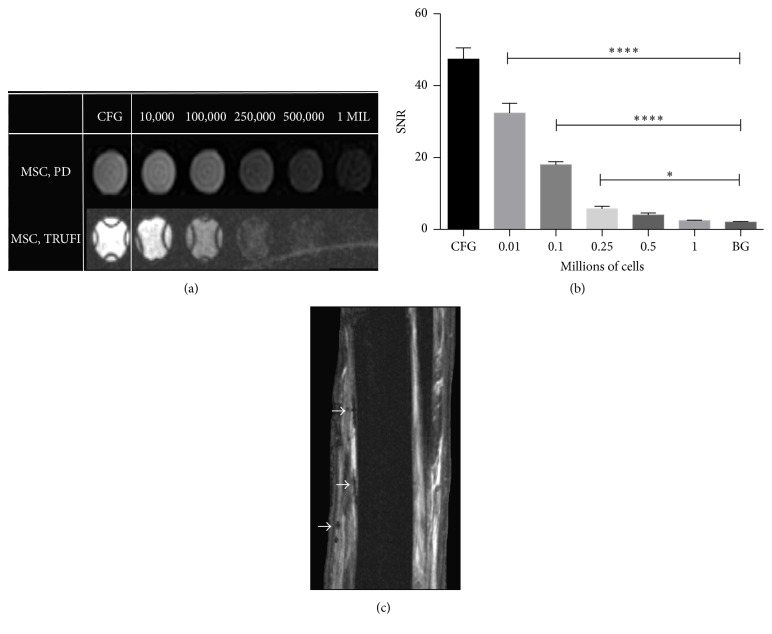
MRI data from cell suspensions in agar gel showing (a) PD (top row) and TRUFI (bottom row) images ranging from cell-free gel (CFG) up to 1 × 10^6^ cells in a 96-well plate. (b) Signal-to-noise ratios (SNR) corresponding to the TRUFI images in (a). (c) Normal tendon following serial injection of 20, 10, 5, and 1 × 10^6^ SPIO-labeled MSCs in order along the palmar aspect of the limb. SPIO-associated signal is only visible in the tissues surrounding the SDFT (arrows) (^*∗∗∗∗*^ *p* < 0.0001, ^*∗*^ *p* < 0.05).

Phantom models indicated that MR signal intensity decreased with increasing labeled cell numbers. All cell concentrations were qualitatively discernible compared to cell-free gel (CFG) on TRUFI and PD-weighted images showing distinct loss of signal (Figure 6(a)). The signal-to-noise ratio (SNR) for all cell concentrations were significantly different as compared to cell-free gel (*p* < 0.0001), but only 10,000 (*p* < 0.0001); 100,000 (*p* < 0.0001); and 250,000 cells (*p* < 0.01) were discernible from background (BG) signal (Figure 6(b)). Cells could not be visualized within normal tendon. Areas of hypointense signal were visible in the paratendinous fascia and subcutaneous tissues and were concentrated near sites of injection following injection into normal tendon (Figure 6(c)).

#### 3.2.2. *In Vivo* MRI Analysis of Intralesional Cell Injection

**Figure 7:**
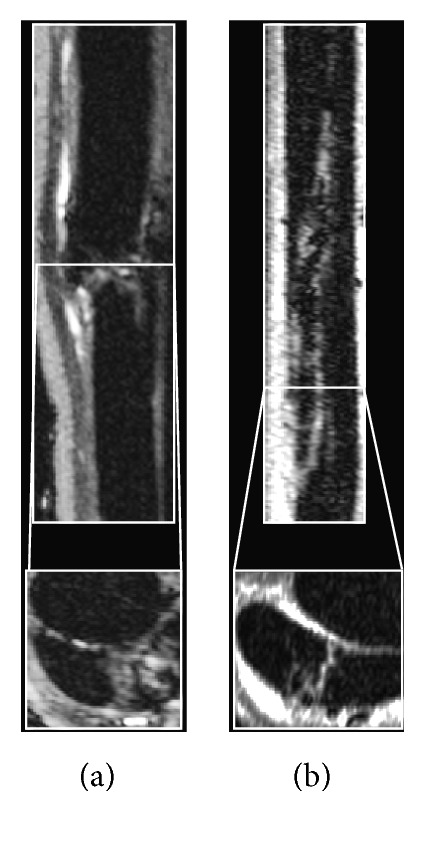
TRUFI images demonstrate SDFT lesions (a) induced immediately prior to and (b) 10 days prior to injection in the sagittal (top) and transverse (bottom) planes.

*(1) Pilot Study*. Iatrogenic injury was localized and consisted of a focal area of fiber disruption and edema at the site of surgical incision in the transverse and sagittal plane. Although a small amount of contrast was produced, contrast was not evident proximally to the site of surgical incision. The extent of cell delivery into the lesion could not be determined (Figure 7(a)). At this time, the model was redesigned such that cells would be injected 10 days following surgery to allow edema to develop and more closely mimic clinical disease. Current studies routinely inject cells 1-2 weeks following iatrogenic injury, which supports the implementation of this timeframe [2, 10, 11, 14, 33, 34].

**Figure 8:**
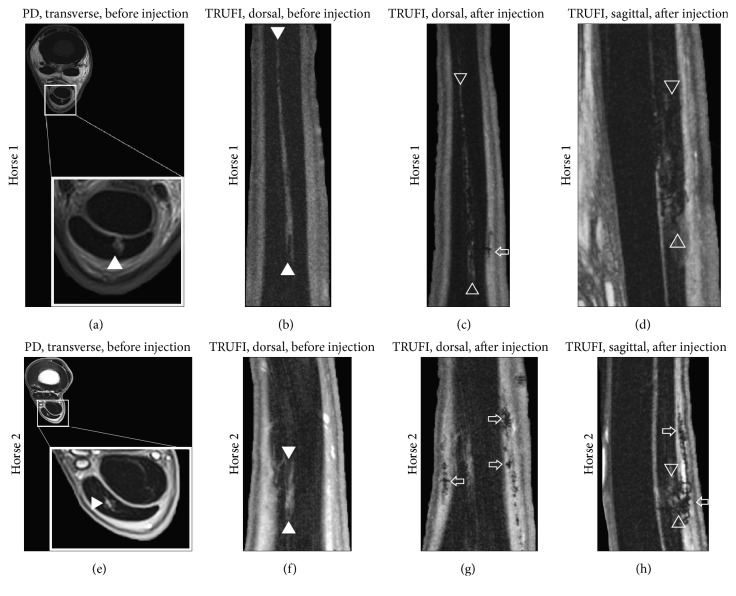
MRI of iatrogenic, SDFT lesions before (a, b, e, f) and after (c, d, g, h) injection of SPIO-treated cells. Solid arrowheads delineate the tendon lesion in preinjection images. Open arrowheads indicate SPIO-treated cells within the tendon lesion after injection. The SPIO-treated cells are seen as small, heterogeneous clusters of dark signal. The open, horizontal arrows indicate SPIO-treated cells distributed throughout the tissue layers surrounding the tendon.

**Figure 9:**
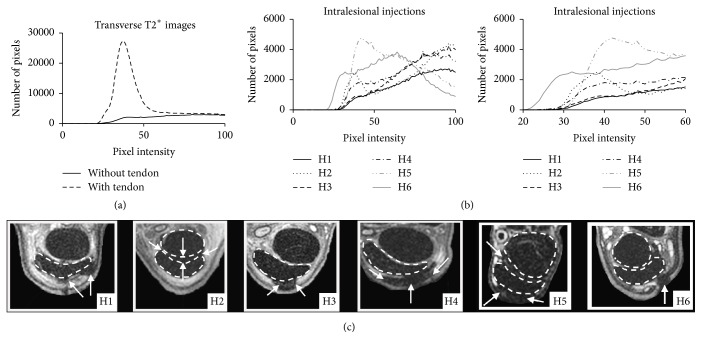
Transverse MRI of SDFT and quantification of low MRI signal as measured on 8-bit, grayscale images. (a) Graph showing summation of low signal intensity pixels before and after subtraction of DDFT and SDFT from transverse images. (b) Summation of low signal intensity pixels in the subcutaneous and surrounding soft tissues in all 6 subjects with a magnified view of the* x*-axis displayed on the right. (c) TRUFI images demonstrating substantial efflux of cells into the paratendinous and surrounding tissues. Arrows indicate hypointense areas of MRI signal representative of SPIO-treated cells. Dotted lines estimate the boundaries of the SDFT and DDFT.

*(2) Model of Tendon Injury*. Prior to injection, tendon injuries were detectable as small, focal areas of hyperintensity localized to the SDFT in the transverse plane (Figures 8(a) and 8(e)) and were visible as longitudinal, linear segments of hyperintense signal in the dorsal plane (Figures 8(b) and 8(f)) in 5/6 subjects. The lesions were measured to be 5.41 ± 0.36 (SE) cm in length, on average. On TRUFI images, clusters of labeled cells were visible as hypointense, signal voids in 6/6 subjects. Coalescing regions of signal void were diffusely present throughout the site of injury, in the paratendinous, subcutaneous, and fibrous scar tissues surrounding the SDF (Figures 8(c), 8(d), 8(g), and 8(h)). Cell retention within the lesions varied greatly, with 1/6 subjects demonstrating little to no cell retention within the lesion (Figures 8(f)–8(h)). Substantial leakage of labeled cells outside of the SDFT was observed. High numbers of low intensity pixel values likely associated with SPIO-labeled cells were quantifiable throughout the tissue surrounding the SDFT and DDFT in 6/6 subjects (Figure 9). Cells were located at 1.89 ± 0.33 (SE) cm proximally and distally to the site of injection and were found at a depth of 3.13 ± 0.40 (SE) mm into the surrounding tissues.

#### 3.2.3. Histology

**Figure 10:**
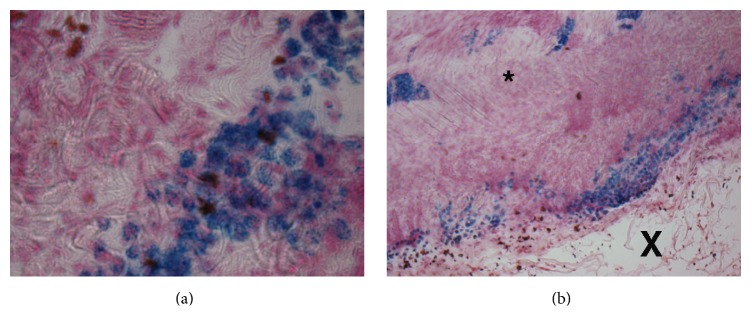
Histology of iron-labeled cells. Tissue sections demonstrate dense areas of Prussian Blue-positive, SPIO-treated MSCs lining the tendon lesion. Markers indicate the tendon lesion (X) and the tendon matrix (*∗*). Images shown are at (a) 10x and (b) 40x.

Tendon lesions were grossly visible as areas of mechanical disruption with little to no fiber density. No scar tissue was present within the lesioned area. The margin surrounding the injury was delineated by dense irregular connective tissue with increased cellularity. The tissue surrounding the lesion was normal with no inflammatory cell infiltrate and demonstrated crimp and fiber patterns characteristic of normal tendon. Prussian Blue-positive, iron-containing cells were detectable within the lesioned area and adherent to the bordering, dense, irregular connective tissue (Figure 10). Few Prussian Blue-positive cells were located within the normal tendon tissue surrounding the lesion.

## 4. Discussion

This study represents a comprehensive evaluation of the characteristics of SPIO-labeled equine MSCs* in vitro*. We aimed to standardize a technique for safely loading equine BM-MSCs with SPIOs and to ensure that MSCs would not be functionally altered by SPIO labeling. A positive correlation was observed between time of incubation with SPIO-treated media and iron load per cell. A previous study demonstrated a linear relationship between SPIO concentration in media and iron load per cell [17]. Increasing time of incubation may provide a more economical alternative to increasing iron load per cell. Our data shows that a higher iron load of 19 pg/cell can be achieved by overnight incubation, but this was detrimental to cell viability. Care must be taken to avoid interference with cell migration and survival when performing cell tracking studies. Subsequent imaging demonstrated robust detectability of clinically relevant cell numbers following only 4 hours of incubation in SPIO-treated media. To ensure maximum cell viability and functionality in future cell tracking studies, we employed a lower iron load of 4 pg/cell for labeling and tracking BM-MSCs although higher loads of 10–12 pg/cell have been validated in prior work [17]. Interestingly, even with this low iron burden, trilineage differentiation showed increased potential for chondrogenesis (ns) and decreased potential for osteogenesis (ns) and an increase in cell proliferation (ns) was noted in SPIO-labeled cells. Previous studies report similar conflicts in trilineage potential and the mechanism of action remains unclear [35–38]. Another study associated changes in cell proliferation with free Fe in the lysosome leading to an increase in cell cycle progression [39]. As such, SPIO-labeled cells should be considered a feasible method for cell tracking* in vivo*, but care should be taken when interpreting results.

The second aim of this study was to establish a clinically relevant, reproducible model for cell detection and tracking in equine tendon injury. The model of iatrogenic injury described by Schramme et al. (2010) has been well characterized on several imaging modalities [28, 29]. However, pathologic tendon is characterized by heterogeneous areas of high and low signal intensity on T2-weighted images, which can be difficult to discern from SPIO-associated signal [14, 15]. SPIOs are nearly undetectable in normal tendon and can only be differentiated in proximity to the subcutaneous tissues where their dipolar effect may disrupt normal tendon borders on MRI. A high field magnet can enhance this effect and is valuable in the interpretation of cell distribution in tissues with low contrast, like tendon.

Previous work with high field MRI showed that ultrasmall SPIOs could effectively be used to track umbilical cord-derived MSCs and BM-MSCs in equine tendons with collagenase defects [40]. Our study implemented a similar imaging approach with the addition of a 15-channel transmit-receive coil, which more closely matches the anatomy of the equine distal limb. High field MRI details pathology more accurately and improves contrast and tissue margins, thus enhancing the ability to monitor cell tracking concurrent with tissue healing [41]. Other studies have attempted to track cells with low field magnets in standing horses [14, 15]. Our choice of equipment and sequences enabled imaging at 0.3 mm slice thickness, approaching a resolution close to that of MR microscopy. This is the first study to achieve this feat using only clinical equipment in the equine distal limb.

This work was designed with the goal of efficient translation into long-term imaging studies that allow simultaneous tracking of cells and tendon healing. Other research relies heavily on T2- and T2^*∗*^-weighted gradient echo sequences [15, 40]. However, T2-weighting enhances the artifacts produced by iron-labeled cells, which may distort the surrounding anatomy and be easily confused with other inhomogeneities in the magnetic field including hemorrhage. Both iatrogenic and collagenase tendon injury models are associated with moderate-to-severe swelling, vascular damage, and induction of the inflammatory cascade. For this reason, the implementation of sequences that offer clear discrimination between hemorrhage and labeled cells is essential. To overcome these challenges, our protocol utilizes three types of MRI sequences: PD-weighted TSE sequences, which enhance pathologic changes in tendon and minimize dephasing caused by labeled cells; TRUFI sequences, which maximize the contrast between signal voids produced by labeled cells while maintaining sufficient SNR and reducing artifact [42]; and T2-weighted sequences, which maximize signal artifacts and are routinely used for imaging SPIOs.

The animal model used in this study is most representative of acute, focal injury to the SDFT and can successfully be used to monitor a large bolus of labeled MSCs after injection by MRI. The use of a mechanical model of injury enhanced detection of MSCs by providing soft tissue contrast within the lesion associated with the absence and disruption of collagen fibers and mild edema. The efflux of cells into the subcutaneous tissues was easily detectable due to the high signal intensity of the fat contrast associated with these surrounding areas. In the future, these cells can easily be tracked and monitored for cell survival and migration into the tendon, as determined by diminution of the hypointense signal and redistribution within the tissues. Longer term* in vivo* tracking studies will also have to consider the effects of biomechanical tendon loads, motion, and gravity on cell movement and distribution.

Our third objective was to characterize the distribution of cells at the time of injection. In contrast to our stated hypothesis, our data suggest that there is a substantial variation in cell distribution among study subjects even though the injection is performed with precise ultrasound triangulation technique. Postinjection T2^*∗*^-weighted images in the transverse plane demonstrated heterogeneous and inconsistent cell localization within the tendon lesions when compared to preinjection images and showed consistent leakage of the labeled cells into the tissue surrounding the SDFT, including the paratenon and subcutaneous tissues, in all subjects.

Histological evaluation confirmed that some cells were also retained within the core lesion. The histologic appearance of the lesioned areas suggests fluid filling that increases interstitial tissue pressure and causes cells to flow retrogradely along the needle surface into the fascial layers surrounding the tendons. It is interesting to note that most equine studies suspend cells in a volume of 1-2 mL for injection into flexor tendon injuries [3, 4, 6, 7, 34], whereas we injected less than 1 mL and still observed poor intralesional cellular retention. The clinical presumption that the majority of a cell bolus is retained within the core lesion is incorrect and further studies should be performed to investigate the degree to which cells migrate within the tendon after injection and if healing is affected by the degree of cell delocalization.

## 5. Limitations

This study performed all imaging and injections postmortem for economical and logistical reasons. Limbs were not injected in weight-bearing conformation, which is common practice in the clinic. However, it is possible that when the flexor tendons are in slack position there is less risk of iatrogenic damage from the incoming fluid and needle stick. If injections are performed in standing position, cells will flow distally to the site of needle placement due to the effects of gravity, as opposed to flowing both proximally and distally when the leg is placed in a horizontal position. The influence of circulation on cell viability within the tendon lesion will also have to be considered in future studies. It is possible that cells engrafted into the subcutaneous tissues may exhibit higher rates of survival than those in the lesion because of higher vascular perfusion and nutrient supply.

Due to the inherent low signal intensity of normal tendon, it is not possible to perform a quantitative evaluation of SPIO signal or to compare the ratio of cells within and surrounding the tendon lesion. However, the images provide a cohesive albeit qualitative assessment regarding the distribution of MSCs following ultrasound-guided, intralesional tendon injections. Although this question has not been raised in the literature in the past, several studies have already begun to investigate cell migration and survival using histology or other methods [2, 10, 11, 14]. The delocalization of cells immediately following cell injection suggests* in vivo* imaging methods will be far superior for analyzing these data. Additionally, since the patterns of cell distribution determined in this study were wider than expected or reported, changes in MSC administration may be needed. However, confirmation of cell location and* in vivo* effect on tendon repair, through tissue biopsy in large animal studies, is needed to better establish a protocol for cellular therapies.

## 6. Conclusion

Although previous reports have determined that local injury retains cells within a small radius of the site of injection, our study shows greater than expected delocalization and that relatively few cells are retained within collagenous tendon compared to surrounding fascia. The theories of MSC mechanism of action may need to consider greater contribution from MSCs outside of collagenous tissue. The regional retention of MSCs may have important implications regarding the healing of injuries. Further work is needed if this is a reality* in vivo* and therefore to determine if directed intralesional delivery of MSCs is as critical as presently thought.

## Figures and Tables

**Table 1:** Parameters for MRI sequences acquired.

Sequence, plane	PD TSE, sagittal	PD TSE, transverse	TRUFI, sagittal and dorsal	T2^*∗*^, transverse	T2 MSE, sagittal
TR (ms)	1830	3020	11.8	695	400
TE (ms)	56	39	5	22.5	4.8, 13, 21.3, 29.6, 37.8
Slice thickness (mm)	2.5	3.0	0.3	2.5	2.5
Flip angle	150	180	28	30	60
FOV (cm)	16 × 16	15 × 15	11 × 16	12.2 × 15	11.4 × 14
Matrix size	320 × 320	320 × 320	352 × 512	260 × 320	260 × 320

## Abbreviations

MRI: Magnetic resonance imaging
SPIO: Superparamagnetic iron oxide nanoparticle
MSCs: Mesenchymal stem cells.
